# Identification of Candidate Biomarkers and Analysis of Prognostic Values in Oral Squamous Cell Carcinoma

**DOI:** 10.3389/fonc.2019.01054

**Published:** 2019-10-18

**Authors:** Guang-zhao Huang, Qing-qing Wu, Ze-nan Zheng, Ting-ru Shao, Xiao-Zhi Lv

**Affiliations:** Department of Oral and Maxillofacial Surgery, NanFang Hospital, Southern Medical University, Guangzhou, China

**Keywords:** competing endogenous RNA, protein-protein interaction, long non-coding RNA, biomarker, oral squamous cell carcinoma

## Abstract

**Objectives:** Oral squamous cell carcinoma (OSCC) is the most common oral cancer with a poor prognosis owing to limited understanding of the disease mechanisms. The aim of this study was to explore and identify the potential biomarkers in OSCC by integrated bioinformatics analysis.

**Materials and Methods:** Expression profiles of long non-coding RNAs (lncRNAs), microRNAs (miRNAs), and messenger RNAs (mRNAs) were downloaded from The Cancer Genome Atlas (TCGA) and differentially expressed RNAs (DERNAs) were subsequently identified in OSCC by bioinformatics analysis. Gene ontology (GO) and Kyoto Encyclopedia of Genes and Genomes (KEGG) pathway analysis were used to analyze DERNAs. Then, the competing endogenous RNA (ceRNA) network was constructed in Cytoscape and the protein -protein interaction (PPI) network was established in the STRING database. We established a risk model to predict the overall survival of OSCC on the basis of DElncRNAs with Kaplan–Meier analysis and combined with logrank p test. Furthermore, we identified potential biomarkers by combining univariate Cox regression with overall survival rate, which were then validated in Gene Expression Omnibus (GEO), OSCC cell lines and OSCC specimens.

**Results:** A total of 1,919 DEmRNAs, 286 DElncRNAs and 111 DEmiRNAs were found to be dysregulated in OSCC. A ceRNA network included 46 DElncRNAs,7 DEmiRNAs and 10 DEmRNAs, and the PPI network included 712 DEmRNAs including 31 hub genes. Moreover, a 7 lncRNAs risk model was established and four genes (CMA1, GNA14, HCG22, HOTTIP) were identified as biomarkers on overall survival in patients with OSCC.

**Conclusions:** This study successfully constructed a ceRNA network and a PPI network which play a crucial role in OSCC. A risk model was established to predict the prognosis, and four DERNAs are revealed with overall survival in patients with OSCC, suggesting that they may be potential biomarkers in tumor diagnosis and treatment.

## Introduction

Oral squamous cell carcinoma (OSCC) is one of the most common oral cancers worldwide (1) and has the most severe impact on the quality of life of patients. The most significant risk factors in OSCC are cigarettes, alcohol and betel nut consumption, which seem to have a synergistic effect. One recent study showed that it may be associated with the infection of human papillomavirus (HPV) (2). Although medical equipment and treatment methods are more advanced in recently years, the 5-year overall survival rate of OSCC remains only 40–50% (3) on account of relatively low responsiveness to treatment, severe drug resistance (4), diagnosis at terminal stage, etc., whereas the 5-year overall survival rate can rise markedly to more than 85% in patients diagnosed with early-staged tumors (5). Therefore, early diagnosis is very important for improving the prognosis of patients with OSCC.

As is known, more than 70% of the human genome can be transcribed to functional products (6). Among them, long non-coding RNAs (lncRNAs), RNA transcripts longer than 200 nucleotides and those with limited protein-coding potential are in the majority. LncRNAs affect various aspects of cellular homeostasis in OSCC, including proliferation, survival, migration, and genomic stability (7). Moreover, cancer-specific lncRNA expression patterns appear more tissue- and stage-specific than those of protein-coding genes, indicating the potential development of lncRNAs as powerful alternative biomarkers and therapeutic targets (8). Meanwhile, recent research demonstrated that lncRNA combined with mRNA biomarkers may improve diagnosis (9). However, there are fewer effective lncRNAs and mRNAs biomarkers used for diagnosis in OSCC.

In 2011, Salmena et al. put forward a competing endogenous RNA (ceRNA) hypothesis (10). MiRNA as a regulatory molecule regulates mRNA expression by targeting the 3'-UTR (11), which typically inhibits the translation and the stability of mRNAs (12). However, lncRNAs can compete with the miRNA binding to the mRNA to regulate gene expression. Recently, increasing evidence indicated that this hypothesis was closely related to the development and initiation of OSCC. For example, lncRNA TUG1 through sponging miR-524-5p to mediate DLX1 expression promotes proliferative and migratory potentials in OSCC (13). In addition, protein-protein interaction (PPI) also plays a crucial role in various cancers. PPI is composed of proteins interacting with each other to participate in various biological processes such as biological signal transmission, regulation of gene expression, energy metabolism, and cell cycle regulation. For example, c-Myc, MMP-2, and GSK3β regulated the expression of MMP9 to accelerate OSCC progression and invasion (14). However, fewer studies have been reported on the ceRNA and PPI networks in OSCC.

In this study, we aim to analyze the differentially expressed profile of non-coding RNAs(ncRNAs) in the OSCC and establish a Cox regression model to predict the overall survival of patients with OSCC. Further, we analyzed and predicted the functions of the ceRNA and PPI networks. This study will contribute to understanding the molecular mechanism and provide new therapeutic targets for OSCC.

## Materials and Methods

### Data Preparation

All primitive data of OSCC (oral cavity, floor of mouth, palate, buccal mucosa, the anterior 2/3 of the tongue, gingiva, and so on) from The Cancer Genome Atlas (TCGA) database (https://portal.gdc.cancer.gov/) were downloaded through GDC Data Transfer Tool, including the RNA-Seq and miRNA-Seq of Transcriptome Profiling and Clinical data. Then, we excluded three samples because of their low-quality clinical data. Finally, 316 OSCC samples and 32 normal control samples were collected in our study. Gene expression profiles of OSCC were obtained from the Gene Expression Omnibus (GEO) database (https://www.ncbi.nlm.nih.gov/geo/), including GSE9844 (26 OSCC samples and 12 normal controls), GSE30784 (167 OSCC samples and 45 normal controls), and GSE74530 (6 OSCC samples and 6 normal controls).

### Collection of OSCC Specimens

A total of 49 pairs of OSCC specimens and normal adjacent tissues (about more than 1.5 cm from the edge of the tumor) were collected at Nanfang Hospital, Southern Medical University (Guangzhou, China), and written informed consent was obtained from all patients. The tissue specimens were stored in RNA Wait and then frozen at −80°C. All tumor tissues and normal adjacent tissues were pathologically confirmed as squamous cell carcinoma and normal tissues, respectively.

### Differentially Expressed Gene Analysis

EdgeR (Version 3.8) package in R software was used to analyze and identify differentially expressed RNAs (DERNAs) and differentially expressed miRNAs (DEmiRNAs) with the thresholds of |log2 (fold change [FC])|>2.0 and FDR (adjusted *P*-value) <0.01 (15). Then, we used the annotation file in GTF format (Homo_sapiens.GRCh38.95.chr.gtf) to identify and annotate differentially expressed long non-coding RNA (DElncRNAs) with the thresholds of |log2FC|>2.0 and FDR <0.01. The heatmap and volcano were constructed by the gplots package in R software.

### Functional Enrichment Analysis

We employed DAVID (https://david.ncifcrf.gov/) to obtain information for Gene Ontology (GO) including biological processes, the cellular component and molecular function. The Kyoto Encyclopedia of Genes and Genomes (KEGG) pathway analysis was used to annotate the potential functions. A significance level of *P* < 0.05 was set as the cutoff criteria and the plots were constructed by the gplots package in R software.

### Protein-Protein Interaction Analysis

The DEmRNAs were enrolled in a protein-protein interaction (PPI) network through the STRING database (https://string-db.org/) with a confidence score >0.9, and the PPI network was visualized in Cytoscape (Version 3.7.1) software. Moreover, genes with degree> = 25 were selected as hub genes. Subsequently, module analysis (16) of the PPI network was performed using the Molecular Complex Detection (MCODE) tool of Cytoscape software, and GO and KEGG analysis of the modules was carried out using the DAVID database.

### Construction of the ceRNA Network

According to the hypothesis of ceRNA, a lncRNA-miRNA-mRNA network was constructed. Relevant miRNA-target data were obtained from the miRcode database (http://www.mircode.org/) (17). Then, the DElncRNA-DEmiRNA interactions were predicted according to the miRcode database. Furthermore, target DEmRNAs were predicted for DEmiRNAs using miRDB (http://www.mirdb.org/) (18), miRTarBase(http://mirtarbase.mbc.nctu.edu.tw/) (19) and TargetScan database (http://www.targetscan.org/) (20), and only the miRNA-mRNA interactions that existed in all the three databases were enrolled in the ceRNA network. Eventually, Cytoscape (Version 3.7.1) was employed to establish the lncRNA-miRNA-mRNA network.

### Cox Risk Regression Establishment and Validation

The lncRNAs raw data were transformed and normalized in a log_2_(x+1) manner (21). OSCC samples were randomly divided into a training set and a validation set. Univariate Cox regression was used to select prognosis-associated genes (*p* < 0.05). Subsequently, we performed Cox regression analysis combined with LASSO to establish a prognostic risk score model, and the penalty regularization parameter lambda (λ) was chosen through cross-validation with an *n*-fold equal to 10 by using the R package glmnet (21). *Lambda.min* was identified to pick out the variables. According to these variables, a stepwise regression was performed to establish the Cox model. Finally, a validation set and Kaplan–Meier survival curves along with a logrank *p* test were applied to validate its accuracy. In addition, receiver operating characteristic (ROC) analysis was used to estimate the predictive power of this signature.

### Cell Culture

The human OSCC cell lines SCC9, SCC15, SCC25, CAL27, and KB and the normal oral epithelial cell line HOK were obtained from the Institute of Antibody Engineering, Southern Medical University (Guangzhou, China). Cells HOK, SCC9, SCC15, and SCC25 were cultured in Dulbecco's modified Eagle's medium F12 (DMEM/F12) (Invitrogen, Carlsbad, CA, USA), CAL27 in α-MEM (Invitrogen, Carlsbad, CA, USA) and KB in RPMI 1640 (Invitrogen, Carlsbad, CA, USA), supplemented with 10% fetal bovine serum (FBS) at 37 °C with 5% CO_2_.

### RNA Extraction and qRT-PCR

Total RNA was extracted from tissues and cells in a TRIzol reagent (Takara) manner. RNA reverse transcription to cDNA was performed with a Reverse Transcription Kit (Vazyme). Quantitative real-time Polymerase Chain Reaction (qRT-PCR) analyses used SYBR Green I (Vazyme) in triplicate. The results were normalized to the expression of GAPDH. The primer sequences are as follows. HCG22 forward primer (5′-3′): CTTCTGCTGCTCCTGCTTCT; reverse primer (5′-3′): ACTCCATCTCTCCAGGTCCC. HOTTIP forward primer (5′-3′): CCTAAAGCCA CGCTTCTTTG; reverse primer (5′-3′): TGCAGGCTGGAGATCCTACT. GNA14 forward primer (5′-3′) CCCA ACAAGATGTGCTTCGC; reverse primer (5′-3′) TCCGTCTTTCCGATCGTTGG. CMA1 forward primer (5′-3′) TCAGCTGTGTGTGGGCAATC; reverse primer (5′-3′) CTTTGCATCCG ACCGTCCAT. GAPDH forward primer (5′-3′): CGCTGAGTACGTCGTGGAGTC; GAPDH reverse primer: (5′-3′) GCTGATGATCTTGAGGCTGTTGTC.

### Western Blot Analysis

Cells and OSCC tissues were lysed in RIPA lysis buffer. According to the procedure, proteins were separated by electrophoresis, transferred to membranes and then sealed with 5% skim milk. The primary antibodies including CMA1 (dilution 1:1,000), GNA14 (dilution 1:1,000) and α-tublin (dilution 1:1,000) were incubated in 4°C for one night. Subsequently, goat anti-mouse and goat anti-rabbit secondary antibodies were incubated for 1 h at room temperature, and finally immunoreactive bands were visualized with a chemiluminescence system.

### Biomarkers Screening and Validation

The status and survival time of OSCC patients were extracted. Subsequently, the mRNA was enrolled in the PPI and ceRNA networks, and lncRNA and miRNA identified in ceRNA were selected for screening biomarkers. We used univariate Cox regression to screen prognostic factors (*p* < 0.05), and those prognostic factors whose expression levels were significantly relevant to patients' overall survival (*p* < 0.01) were selected as primitive biomarkers. Ultimately, the gene expression profile from the GEO and OSCC cell lines and tissues were used to verify these biomarkers. In addition, a combination of two or more biomarkers was performed to predict OSCC overall survival according to the gene expression in TCGA.

### Statistical Analysis

Statistical analyses were performed using SPSS23.0 software (IBM). The differences between groups were tested using a two-tailed Student's *t*-test. The survival analysis between two groups was conducted by logrank test. *P*-values <0.05 were considered statistically significant. Differences were considered significant if ^*^p < 0.05; ^**^p < 0.01; ^***^p < 0.001; or ^****^p < 0.0001.

## Results

### Identification of DEmRNA, DEmiRNAs, and DElncRNAs

**Figure 1:**
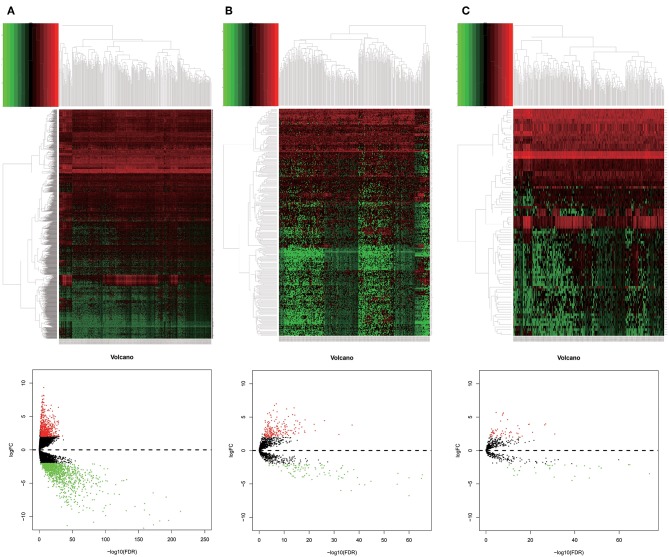
Distributions of differentially expressed genes in oral squamous cell carcinoma (OSCC) (|log2FC| >2.0 and adjusted *P*-value < 0.01) between 316 tumor tissues and 32 normal tissues. The ascending normalized expression level in the heatmaps is colored from green to red. Red means gene upregulation, green indicates downregulation and black means normal expression. Furthermore, each column represents a sample and each row represents a differentially expressed gene. The heatmaps plot 1919 DEmRNAs **(A)**, 192 DElncRNAs **(B)**, and 111 DEmiRNAs **(C)**. Similar with heatmaps, red stands for upregulations, green stands for downregulation and black stands for normal expression in volcanoes. Each point represents a gene.

RNA expression profiles and corresponding clinical data of 316 OSCC patients and 32 normal controls were downloaded from TCGA database. With the cut-off criteria unified, |log2FoldChange|>2 and FDR <0.01, 1919 DEmRNAs (673 upregulated and 1246 downregulated, Figure 1A), 286 DElncRNAs (192 upregulated and 94 downregulated, Figure 1B) and 111 DEmiRNAs (61 upregulated and 50 downregulated, Figure 1C) were sorted out.

### Functional Analysis of DERNAs

**Figure 2:**
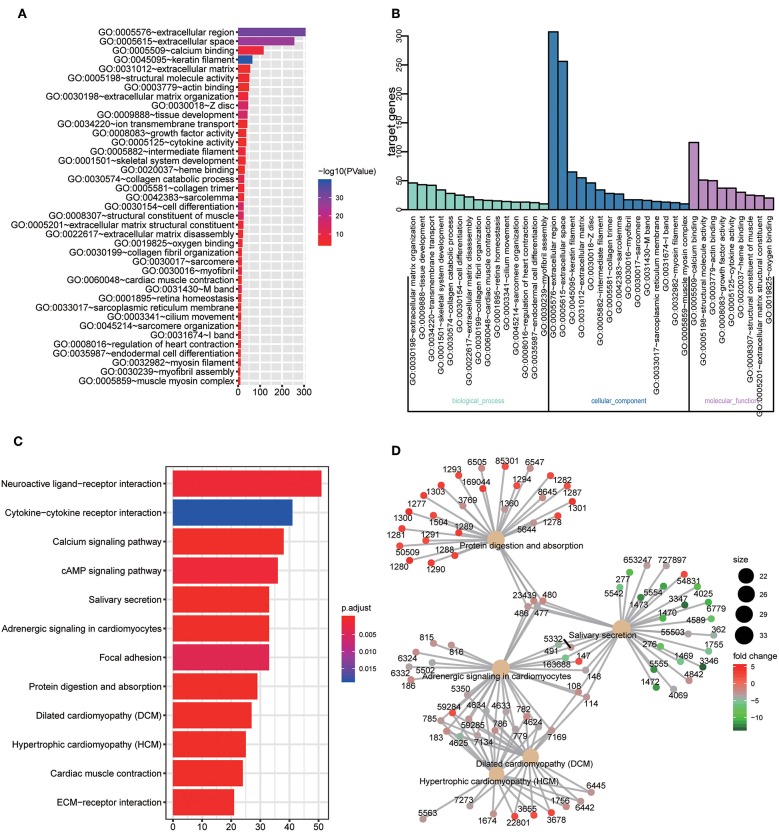
GO and KEGG pathway analysis of DERNAs. **(A)** The numbers of genes enriched in each GO category. The y axis represents the GO categories including the biological process, cellular component and molecular function, and the x axis represents the enrichment score. Furthermore, the color stands for *p*-value. **(B)** GO analysis contains the biological process (BP, in green), cellular component (CC, in blue) and molecular function (MF, in purple). The y axis represents the target gene and the x axis represents the biological process. **(C)** The most important pathways in DERNAs. The y axis represents the pathways and the x axis represents enriched gene numbers, and the color means adjust *P*-value. **(D)** The Netplot of KEGG pathways, mean enrichment of genes in different pathways. The number adjacent to nodes stands for gene ID.

GO and KEGG enrichment analysis were used to explore the potential function of DERNAs. The results indicated that these genes were mainly enriched in tissue development, cell differentiation and calcium binding (Figures 2A,B). Moreover, the majority of genes were located in the extracellular region. In addition, KEGG pathway analyses demonstrate that the most significant pathways were the calcium signaling, protein digestion and absorption and focal adhesion pathways (Figures 2C,D).

### Protein-Protein Interaction (PPI) Network Analysis

**Figure 3:**
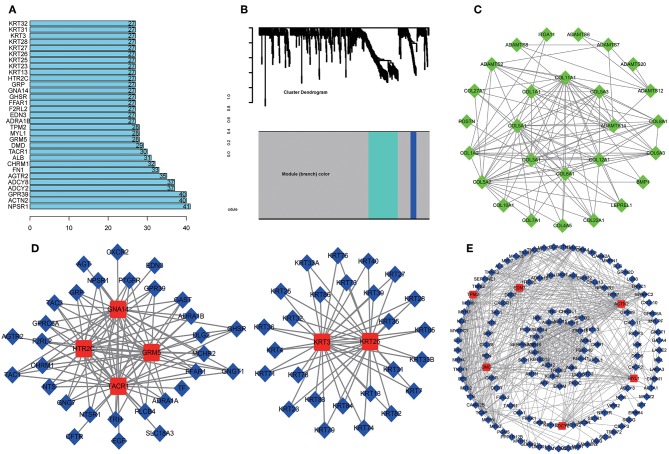
Protein-protein network (PPI) based on the DEmRNAs with a combined score was >0.90. **(A)** 31 hug genes were listed because the degree was >25 in the PPI network. **(B)** Module analysis of DEmRNA enrolled in PPI network with the criterion cut. Height = 0.8, min size = 10. Same color means it belongs to the same module. And 3 modules were visualized in Cytoscape **(C–E)**. The connection between the nodes indicates the potential interaction between different mRNA, and red represents the hub gene in the PPI network. Meanwhile, GO and KEGG analysis of 3 modules was performed in DAVID (Tables 1–3).

A total of 712 proteins and 3,181 edges were selected in the PPI network. The confidence score is >0.9. A total of 31 hub genes were selected from PPI network with degree> = 25 (Figure 3A). Among them, 6 mRNAs including GNA14, GRM5, KRT3, KRT26, TACR1, and HTR2C were related to overall survival. In addition, according to module analysis, three modules were identified in the PPI network (Figures 3B–E). Surprisingly, most hub genes, including all of these associated with overall survival (Supplementary Figure 1), were enriched in module 2 indicating that module 2 plays a vital role in the PPI network. According to the GO terms analysis, three modules related to cell adhesion, tissue development, and protein binding played a crucial role in cancer. With respect to KEGG enrichment analysis, modules 1 and 2 regulated metabolic pathways such as protein digestion and absorption (Tables 1, 2). Moreover, modules 1 and 3 were significantly relevant to the PI3K-Akt signaling and calcium signaling pathways, which were associated with the occurrence and development of tumors (Table 3).

**Table 1:** Module 1 GO and KEGG pathways analysis.

	**ID**	**GO-term**	**Count**	***p*-value**
Biological process	GO:0030199	Collagen fibril organization	10	5.07E-22
	GO:0030198	Extracellular matrix organization	13	7.24E-22
	GO:0043933	Protein-containing complex subunit organization	11	2.22E-08
	GO:0071230	Cellular response to amino acid stimulus	5	3.58E-08
	GO:0043588	Skin development	7	1.08E-07
Molecular function	GO:0048407	Platelet-derived growth factor binding	5	1.33E-11
	GO:0046332	SMAD binding	3	0.0003
	GO:0005201	Extracellular matrix structural constituent	3	0.0003
	GO:0004222	Metalloendopeptidase activity	3	0.00059
	GO:0002020	Protease binding	3	0.00088
Cellular component	GO:0005581	Collagen trimer	9	5.36E-17
	GO:0062023	Collagen-containing extracellular matrix	9	1.85E-15
	GO:0031012	Extracellular matrix	10	2.75E-15
	GO:0005583	Fibrillar collagen trimer	6	4.20E-15
	GO:0044420	Extracellular matrix component	7	5.33E-14
	**Pathways**	**Description**		
KEGG pathways	hsa04974	Protein digestion and absorption	9	9.42E-18
	hsa04512	ECM-receptor interaction	4	1.03E-06
	hsa04510	Focal adhesion	4	2.15E-05
	hsa05165	Human papillomavirus infection	4	7.32E-05
	hsa05146	Amoebiasis	3	7.32E-05

**Table 2:** Module 2 GO and KEGG pathways analysis.

	**ID**	**GO-term**	**Count**	***p*-value**
Biological process	GO:0050896	Response to stimulus	214	1.08E-23
	GO:0065008	Regulation of biological quality	127	2.60E-18
	GO:0010033	Response to organic substance	110	5.34E-18
	GO:0042221	Response to chemical	137	1.32E-17
	GO:0043062	Extracellular structure organization	38	1.58E-17
Molecular function	GO:0005488	Binding	242	1.86E-10
	GO:0005102	Signaling receptor binding	64	1.86E-10
	GO:0030545	Receptor regulator activity	33	8.42E-10
	GO:0004252	Serine-type endopeptidase activity	21	8.42E-10
	GO:0048018	Receptor ligand activity	31	3.00E-09
Cellular component	GO:0044421	Extracellular region part	92	1.22E-30
	GO:0005576	Extracellular region	123	1.22E-30
	GO:0005615	Extracellular space	76	5.72E-25
	GO:0031012	Extracellular matrix	30	1.14E-13
	GO:0044420	Extracellular matrix component	14	4.21E-10
	**Pathways**	**Description**		
KEGG pathways	hsa04080	Neuroactive ligand-receptor interaction	23	3.49E-08
	hsa04657	IL-17 signaling pathway	12	6.60E-06
	hsa04512	ECM-receptor interaction	11	9.63E-06
	hsa00830	Retinol metabolism	10	9.63E-06
	hsa04610	Complement and coagulation cascades	10	3.33E-05

**Table 3:** Module 3 GO and KEGG pathways analysis.

	**ID**	**GO-term**	**Count**	***p*-value**
Biological process	GO:0003012	Muscle system process	41	1.28E-49
	GO:0006936	Muscle contraction	39	4.87E-49
	GO:0030049	Muscle filament sliding	24	1.43E-40
	GO:0070252	Actin-mediated cell contraction	26	6.47E-38
	GO:0030029	Actin filament-based process	31	1.35E-26
Molecular function	GO:0008092	Cytoskeletal protein binding	33	1.23E-21
	GO:0003779	Actin binding	25	1.28E-20
	GO:0008307	Structural constituent of muscle	13	4.89E-18
	GO:0051015	Actin filament binding	14	3.53E-13
	GO:0005198	Structural molecule activity	19	9.42E-10
Cellular component	GO:0044449	Contractile fiber part	34	1.75E-42
	GO:0030017	Sarcomere	33	4.42E-42
	GO:0099512	Supramolecular fiber	35	5.34E-25
	GO:0031674	I band	19	3.53E-22
	GO:0015629	Actin cytoskeleton	26	3.53E-22
	**Pathways**	**Description**		
KEGG pathways	hsa05414	Dilated cardiomyopathy (DCM)	14	2.95E-16
	hsa05410	Hypertrophic cardiomyopathy (HCM)	13	1.66E-15
	hsa04261	Adrenergic signaling in cardiomyocytes	15	1.66E-15
	hsa04260	Cardiac muscle contraction	11	1.03E-12
	hsa04020	Calcium signaling pathway	12	2.48E-10

### Construction of ceRNA Network in OSCC

**Figure 4:**
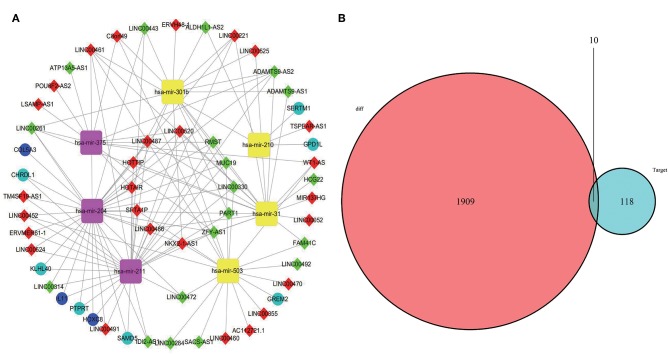
CeRNA network was visualized in Cytoscape **(A)**. The yellow represents miRNA upregulation and purple downregulation. The red means lncRNA upregulation and green downregulation. The mazarine indicates mRNA upregulation and blue downregulation. Gray edges indicate lncRNA-miRNA-mRNA interactions. **(B)** Venn diagram of DEmiRNAs might target DEmRNAs.

A total of 46 lncRNAs, 10 mRNAs and 7 miRNAs were enrolled in the ceRNA network (Figure 4A). We employed miRcode to predict the potential miRNA target by DElncRNAs. As a result, 46 of 286 DElncRNAs and 7 of 111 DEmiRNAs formed 119 lncRNA-miRNA pairs (Table 4). Then, miRDB, miRanda and TargetScan were used to predict the miRNA-mRNA pairs. Only the miRNA-mRNA interactions that exist in all three databases were brought into the ceRNA network (Table 5). Finally, there were 10 DEmRNAs could target to the 7 miRNAs (Figure 4B). Therefore, according to Table 6, the ceRNA network including 46 lncRNAs, 10 mRNAs and 7 miRNAs was completely constructed, and the lncRNA-miRNA-mRNA network was visualized in Cytoscape (Version 3.7.1).

**Table 4:** The miRcode database revealed interactions between DElncRNAs and DEmiRNAs.

**miRNA**			**lncRNA**		
hsa-mir-204	PART1	LINC00314	LINC00221	MUC19	LINC00487
	SFTA1P	HOTAIR	LINC00452	LINC00443	ERVMER61-1
	LINC00486	IDI2-AS1	ZFY-AS1	LINC00472	LINC00330
	TM4SF19-AS1	ADAMTS9-AS2	HOTTIP	LINC00461	LINC00491
	NKX2-1-AS1	RMST	LINC00520	LINC00524	LINC00261
hsa-mir-210	ERVMER61-1	LINC00461	ALDH1L1-AS2	TSPEAR-AS1	LINC00443
	PART1	LINC00314	LINC00221	MUC19	LINC00487
	SFTA1P	HOTAIR	LINC00452		
hsa-mir-211	LINC00486	IDI2-AS1	ZFY-AS1	LINC00472	LINC00330
	TM4SF19-AS1	ADAMTS9-AS2	HOTTIP	LINC00461	LINC00491
	NKX2-1-AS1	RMST	LINC00520	LINC00524	LINC00261
	LINC00525	PART1	LINC00221	MUC19	HOTAIR
hsa-mir-301b	LINC00443	ERVH48-1	LINC00330	ADAMTS9-AS1	ADAMTS9-AS2
	HOTTIP	ALDH1L1-AS2	NKX2-1-AS1	C8orf49	RMST
	LINC00261	LINC00525	PART1	WT1-AS	LINC00221
	MUC19				
hsa-mir-31	LINC00487	HCG22	FAM41C	LINC00486	MIR137HG
	ZFY-AS1	ADAMTS9-AS1	ADAMTS9-AS2	HOTTIP	LINC00461
	NKX2-1-AS1	RMST	LINC00520	LINC00052	LINC00261
	WT1-AS	MUC19	HOTAIR	ZFY-AS1	POU6F2-AS2
hsa-mir-375	ATP13A5-AS1	LSAMP-AS1	ADAMTS9-AS2	C8orf49	RMST
	LINC00520	LINC00261	SFTA1P	LINC00355	LINC00470
	WT1-AS	MUC19			
hsa-mir-503	LINC00452	SACS-AS1	FAM41C	LINC00472	LINC00460
	LINC00284	LINC00330	AC112721.1	LINC00461	LINC00492
	NKX2-1-AS1	C8orf49	LINC00520		

**Table 5:** The miRDB, miRanda, and TargetScan database revealed interactions between DEmiRNAs and DEmRNAs.

**miRNA**	**mRNA**
hsa-mir-204	COL5A3 PTPRT KLHL40
	SAMD5 CHRDL1 HOXC8 IL11
hsa-mir-210	GPD1L SERTM1
hsa-mir-211	PTPRT IL11 KLHL40
	HOXC8 CHRDL1 SAMD5
hsa-mir-503	GREM2

**Table 6:** lncRNA-miRNA-mRNA network.

**miRNA**	**mRNA**	**lncRNA**
hsa-mir-204	COL5A3 PTPRT KLHL40 SAMD5 CHRDL1 HOXC8 IL11	PART1 LINC00314 LINC00221 MUC19 LINC00487 SFTA1P HOTAIR LINC00452 LINC00443 ERVMER61-1 LINC00486 IDI2-AS1 ZFY-AS1 LINC00472 LINC00330 TM4SF19-AS1 ADAMTS9-AS2 HOTTIP LINC00461 LINC00491 NKX2-1-AS1 RMST LINC00520 LINC00524 LINC00261
hsa-mir-210	GPD1L SERTM1	TSPEAR-AS1 LINC00461 ALDH1L1-AS2 PART1 LINC00314 LINC00221 MUC19 LINC00487 SFTA1P HOTAIR LINC00452 LINC00443 ERVMER61-1
hsa-mir-211	PTPRT IL11 KLHL40 HOXC8 CHRDL1 SAMD5	LINC00486 IDI2-AS1 ZFY-AS1 LINC00472 LINC00330 TM4SF19-AS1 ADAMTS9-AS2 HOTTIP LINC00461 LINC00261 LINC00491 NKX2-1-AS1 RMST LINC00520 LINC00524 LINC00525 PART1 LINC00221 MUC19 HOTAIR
hsa-mir-503	GREM2	LINC00452 SACS-AS1 FAM41C LINC00472 LINC00460 LINC00284 LINC00330 AC112721.1 LINC00461 LINC00492 NKX2-1-AS1 C8orf49 LINC00520

### Establishment and Validation of Cox Regression Model

**Figure 5:**
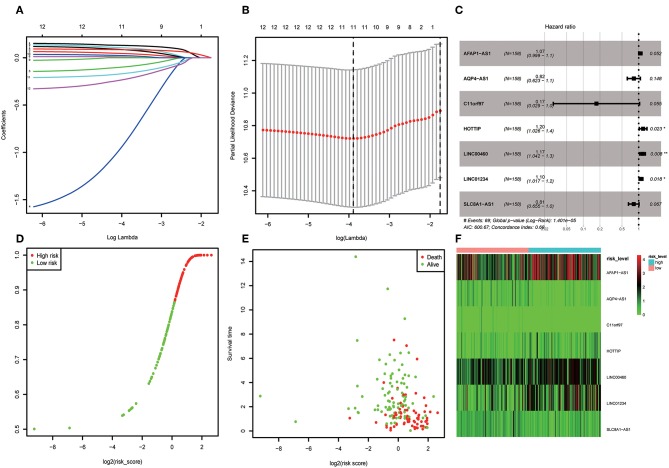
**(A)** LASSO coefficient profiles of the genes associated with the overall survival of OSCC. **(B)** Partial likelihood deviance was plotted vs. log(c). The vertical dotted line indicates the lambda value with the minimum error and the largest lambda value, where the deviance is within one SE of the minimum. **(C)** Forest map based on the risk score model. Left vertical dotted line indicates protective genes and right risk genes. **(D)** Overview of risk evaluation models. The y axis represents percentage and x axis log2 (risk score). **(E)** The scatter diagram based on survival time and log2 (risk score). The red means death and green life. The higher log2 (risk score) is, the shorter the time survival. **(F)** Differentially expressed lncRNAs which were enrolled in the risk model heatmap.

A total of 316 OSCC samples were randomly divided into a training set and validation set. Subsequently, combined univariate Cox regression with a LASSO Cox regression model along with 10-fold cross-validation, 11 variables (AC073130.1, AFAP1-AS1, AQP4-AS1, C11orf97, HOTTIP, HOXA11-AS, LINC00460, LINC01234, LINC01391, SLC8A1-AS1, and SRGAP3-AS2) were identified (Figures 5A,B). Furthermore, a stepwise regression was performed according to the 11 lncRNAs. Consequently, 7 lncRNAs including AFAP1-AS1, AQP4-AS1, C11orf97, HOTTIP, LINC00460, LINC01234, and SLC8A1-AS1 were harvested in Cox regression. Risk score = (0.06844^*^AFAP1-AS1)–(1.7559^*^ *C11orf97)* +(0.18417^*^HOTTIP)+(0.16046^*^ *LINC00460)*+ (0.09473^*^LINC01234)–(0.20486^*^SLC8A1-AS1)- (0.20076^*^AQP4-AS1) (Figure 5C). Afterwards, the OSCC patients were divided into high risk and low risk group based on median value of Cox regression model. The distribution of the risk score along with the corresponding survival data and the 7 lncRNAs expression demonstrated that the high-risk OSCC patients tended to experience shorter survival time, and low-risk patients were opposite (Figures 5D,E). Correspondingly, the protective genes in high risk group expression level were lower than low group. On the contrary, risky genes were higher in high-risk group (Figure 5F).

Kaplan–Meier curves along with logrank *p* test were used to evaluate its accuracy in the training and validation set. According to the risk formula, obvious differences in survival analysis were determined in high- and low-risk groups (Supplementary Figures 2A,B). Meanwhile, stratified analysis in disparate grades was further carried out and indicated that risk level was relevant to prognosis (Supplementary Figures 2C,D). Subsequently, the ROC was plotted, and its area Under the Curve (AUC) is 0.776 (Supplementary Figure 2E).

### Screening Biomarkers

**Figure 6:**
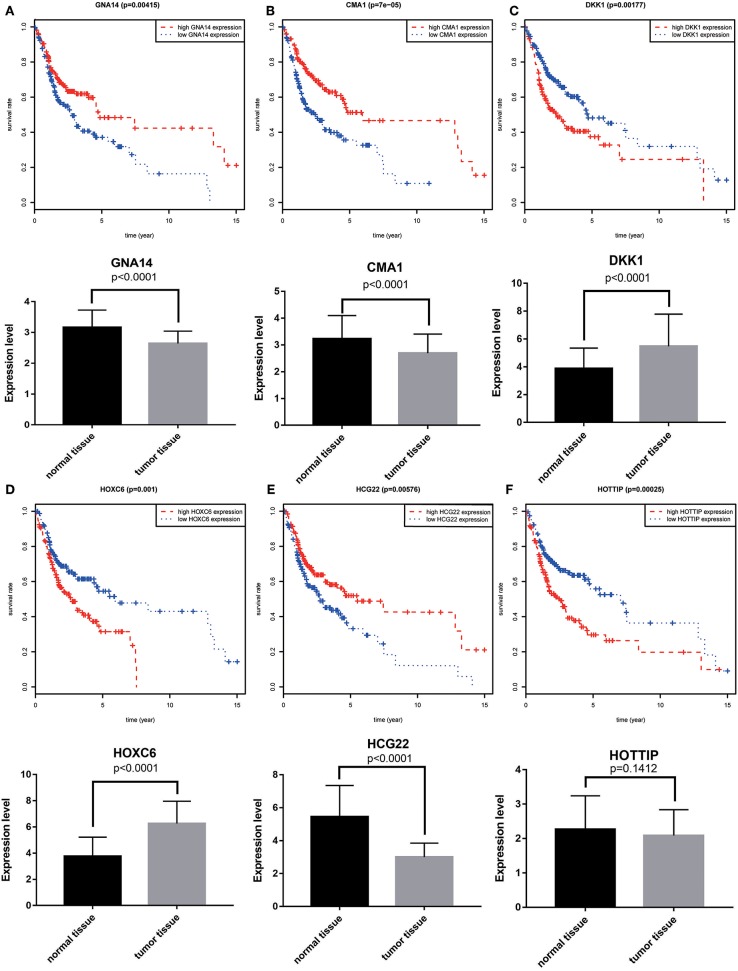
Combining Kaplan–Meier survival analysis with univariate Cox regression to screen biomarkers in OSCC patients. Kaplan–Meier survival curves and GEO gene expression profiles for the three protective genes, GNA14 **(A)**, CMA1 **(B)** and HCG22 **(C)**, and risky genes DKK1 **(D)**, HOXC6 **(E)**, and HOTTIP **(F)** were associated with overall survival in OSCC. GNA14:G protein subunit alpha 14; CMA1:chymase 1; DKK1: dickkopf WNT signaling pathway inhibitor 1; HOXC6:homeobox C6; HOTTIP:HOXA distal transcript antisense RNA; HCG22:HLA complex group 22.

A total of 6 genes (GNA14, CMA1, DKK1, HOXC6, HCG22, and HOTTIP) were identified as primitive biomarkers. The results of univariate Cox regression indicated that there were 36 mRNAs (Table 7-3) and 6 lncRNAs (Table 7-1) regarded as prognostic factors (*p* < 0.05). However, none of the miRNAs were related to prognosis (Table 7-2). Meanwhile, following the combined Kaplan–Meier curves with logrank *p* test, the genes were clearly associated with overall survival (*p* < 0.01) and selected as primitive biomarkers. Finally,4 mRNAs (Figures 6A–D) and 2 lncRNAs (Figures 6E,F) were identified. Subsequently, GEO expression profiles were used to verify the 6 biomarkers. As expected, most of the biomarker expression levels in GEO were consistent with TCGA. However, lncRNA HOTTIP upregulated in TCGA and there was no difference in GEO.

**Table 7-1:** lncRNA univariate Cox regression results.

**lncRNA**	**HR**	***p*-value**
LINC01234	1.132608781	0.000664542
AC073130.1	1.283657314	0.007536904
HCG22	0.76621593	0.020101083
HOTTIP	1.190591243	0.02547972
AFAP1-AS1	1.07265706	0.037909467
LINC01322	1.090081006	0.040038896

**Table 7-2:** miRNA univariate Cox regression results.

**miRNA**	**HR**	***p*-value**
hsa-mir-503	0.912139652	0.187845207
hsa-mir-301b	1.054398906	0.446577624
hsa-mir-31	1.039526476	0.334137026
hsa-mir-204	1.077655651	0.08420409
hsa-mir-211	1.034251589	0.67807554
hsa-mir-375	0.948826626	0.186073347
hsa-mir-210	1.004057662	0.941092685

**Table 7-3:** mRNA univariate Cox regression results.

**mRNA**	**HR**	***p*-value**	**mRNA**	**HR**	***p*-value**
CMA1	0.84609833	0.000233691	SLCO1B1	1.106636606	0.031730512
RSPO1	0.841607027	0.00031998	GLP1R	0.873802152	0.031856886
DKK1	1.113114821	0.001466491	TMOD1	1.083680927	0.032298388
GRIA3	0.875364383	0.002180697	ADCY2	1.074643204	0.034551472
IL17A	0.836798749	0.002950362	HOXC6	1.152748928	0.035502816
IL17F	0.770775988	0.003080682	CDKN2A	0.947072266	0.036677585
ALB	1.097351002	0.009178569	HIST1H2AG	1.160899705	0.036710592
PLAU	1.227694927	0.010331743	PKLR	0.908396028	0.037664026
HAO2	0.835004357	0.010788042	NPSR1	1.087738487	0.038197924
GNA14	0.82424831	0.011610204	ACAN	0.887171624	0.038226114
EGF	1.104743198	0.01450506	ENO2	1.137767875	0.038983131
RSPH4A	0.83254099	0.018772383	HBA2	0.914609549	0.0437109
ENPP3	0.874588953	0.023424344	NRG3	0.868960968	0.043744345
GRM5	0.83886418	0.023657185	PCK1	1.100095409	0.044003417
AGTR2	1.186356377	0.024809836	DMP1	1.128487651	0.044165512
MLXIPL	1.082210876	0.028255755	SPTB	1.080475995	0.045995825
PLIN1	1.104355635	0.030146844	AKR1C3	1.073842523	0.047306782
IL13RA2	0.916862105	0.030234187	OLFM4	1.053069233	0.048107359

### Validation for Biomarkers

**Figure 7:**
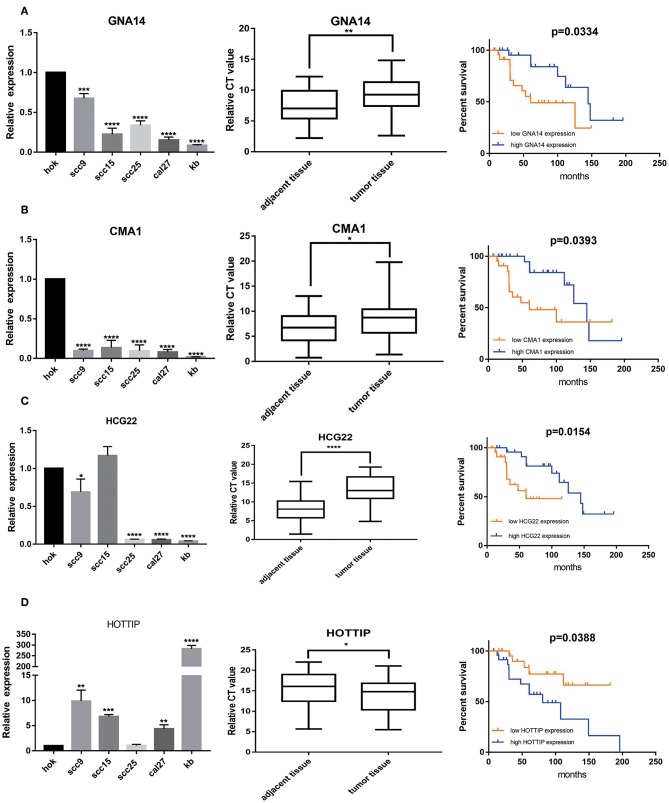
The expression levels of 2 mRNAs, GNA14 **(A)** and CMA1 **(B)**, and 2 lncRNAs HCG22 **(C)**, and HOTTIP **(D)** in 5 OSCC cell lines and 49 pairs adjacent tissues and tumor tissues. HOK is used as control. Then, Kaplan–Meier analysis along with logrank p was used to compare the survival of the low expression group and the high expression group. However, mRNA levels of DKK1 **(E)** and HOXC6 **(F)** showed no difference in OSCC tissues. ^*^p < 0.05; ^**^p < 0.01; ^***^p < 0.001; ^****^p < 0.0001.

A total of 2 mRNAs (GNA14 and CMA1) and 2 lncRNAs (HCG22 and HOTTIP) were differentially expressed in OSCC cell lines and OSCC tissues. Our results revealed that CMA1, GNA14, and HCG22 had low expression in OSCC cell lines and tissues. However, lncRNA HOTTIP was highly expressed in tumor tissues compared with adjacent normal tissues. Meanwhile, Kaplan- Meier analysis suggested that low expression of GNA14, CMA1, and HCG22 were related to poor survival (Figures 7A–C). High HOTTIP expression showed obviously poorer overall survival than those with low HOTTIP expression (Figure 7D). Unfortunately, there was no difference for DKK1 and HOXC6 in OSCC tissues, indicating that the 2 mRNAs might not be biomarkers in OSCC (Supplementary Figure 3A). Meanwhile, the protein levels of CMA1 and GNA14 were detected in OSCC cell lines and tissues (Supplementary Figure 3B). Finally, a combination of these biomarkers can predict overall survival better (Supplementary Figures 3A,B).

## Discussion

In the past decades, the 5-year survival rate of OSCC has remained low (3) in spite of advances in surgical treatment and radiotherapy. Hence, it is vital to find and identify biomarkers for early diagnosis and prognosis of OSCC.

In this study, a total of 1,919 DEmRNAs, 286 DElncRNAs and 111 DEmiRNAs were identified. GO analysis revealed that the function of DERNAs is mainly associated with tissue development, cell differentiation and calcium binding, which play a vital role in tumorigenesis. In addition, KEGG pathways analysis showed that DERNAs mainly enriched in the protein digestion and absorption, calcium signaling and focal adhesion pathways. Among these pathways, the calcium signaling and focal adhesion pathways are significantly associated with cancers. For example, abnormal Ca2+ level is related to glioblastoma and gastric adenocarcinoma (22, 23). In addition, research shows that focal adhesion is relevant to therapy resistance and plays a vital role in carcinogenesis, tumor progression and metastasis (24).

In the present study, 712 mRNAs were enrolled in the PPI network, and module analysis was performed. The majority of these genes were relevant to the PI3K-Akt signaling and calcium signaling pathways, which are associated with occurrence and development of tumors (25, 26). Meanwhile, the PI3K-Akt signaling pathway also played a significant role in OSCC (27). Six hub genes associated with overall survival—GNA14, GRM5, KRT3, KRT26, TACR1, and HTR2C—were enriched in module 2, indicating that module 2 plays a vital role in the PPI network and OSCC prognosis. To our surprise, GNA14 was identified as a potential biomarker by PCR assay and as a hub gene by bioinformatics analysis, which indicated that GNA14 may play a crucial role in OSCC carcinogenesis.

In our ceRNA network, 2 lncRNAs (HOTIP,HCG22) were identified as prognostic biomarkers. Recently, increasing studies showed that aberrant expression of HOTTIP is associated with various cancers. For instance, knockdown of HOTTIP suppresses growth and invasion and induces apoptosis of oral tongue squamous carcinoma cells (28). However, HOTTIP expression has no difference in GEO, which may be associated with racial difference. In addition, bioinformatics analysis confirmed that HCG22 is differentially expressed in various cancers (29), and the mechanism in OSCC should be researched.

Because of poor prognosis and high recurrence, we constructed a risk score formula by comprehensive analysis of lncRNA to predict overall survival in OSCC. Finally, 7 lncRNA were enrolled in Cox regression and it can predict overall survival accurately. In oral cancer, overexpression of the lncRNA AFAP1-AS1 is associated with the proliferation, invasion and survival of tongue squamous cell carcinoma via the Wnt/β-catenin signaling pathway. LINC00460/EZH2/ KLF2 and LINC00460/miR-149-5p/CUL4A crosstalk serve as critical effectors in CRC tumorigenesis and progression (30). Chen et al. showed that LINC01234 expression was significantly upregulated in gastric cancer tissues and functioned as a ceRNA to regulate CBFB expression by sponging miR-204-5p (31). However, there is no report on lncRNA C11orf97, SLC8A1-AS1, and AQP4-AS1. Whether these lncRNAs play important roles in the development and progression of OSCC remains to be further investigated. Meanwhile, univariate Cox regression and multivariate Cox regression analysis suggested that HOTTIP was related to prognosis, which indicated that HOTTIP was regarded as an independent prognostic biomarker.

Eventually, four biomarkers including GNA14, CMA1, HOTTIP, and HCG22 were selected as biomarkers in OSCC by combining comprehensive analysis with PCR validation. Neuhaus J et al. indicated that aberrant CMA1 expression was found in Prostate Cancer (32). In addition, GNA14 was identified as biomarkers and a hub gene, suggesting that GNA14 was obviously relevant to OSCC. Our analysis shows that lncRNA HOTTIP and HCG22 are also biomarkers of OSCC. HOTTIP and HCG22 can interact with hsa-mir-21 in the ceRNA network, which promote oral cancer invasion via the Wnt/β-catenin pathway (33). Accordingly, exploring their mechanism in OSCC may provide new therapeutic targets. Furthermore, DKK1 and HOXC6 were excluded because there was no difference in mRNA level in OSCC tissues. The reasons may be racial difference or limited sample size.

We successfully constructed the ceRNA network, which reveals the potential mechanisms between lncRNA and mRNA. It may provide a new vision on therapeutic targets in OSCC by exploring the underlying mechanisms. At the same time, we also constructed a 7 lncRNA risk score model which is positively associated with overall survival in OSCC. We can put forward reasonable therapy at an appropriate time according to the risk model. Meanwhile, determining revisiting patients and follow-up improve the overall survival in OSCC based on risk level. Eventually, based on the 4 biomarkers, it may be beneficial to realize early-stage diagnosis and provide new therapeutic targets in OSCC.

Though the study might have crucial clinical importance, we still need to consider several limitations. First, in terms of sample numbers, both the TCGA database and clinical specimens which were collected at Nanfang Hospital are far from inadequate. We need to collect more information to continue verifying its accuracy. Second, the information from TCGA may show bias. Although we have validated it in cell lines and clinical specimens, we should continue to do further research. Third, the function and mechanism of biomarkers in OSCC need to be further studied *in vivo* and *in vitro*.

## Conclusion

In summary, our study identifies that 2 mRNAs and 2 lncRNAs might be novel important prognostic factors and potential treatment targets for OSCC. Furthermore, we established a disordered lncRNA-miRNA-mRNA ceRNA network which is beneficial to understanding the relationship between lncRNA and mRNA and provides efficient strategies for subsequent functional studies of lncRNAs. Meanwhile, construction of the PPI network provides a new vision for OSCC treatment, and the risk score model is helpful for improving the overall survival in OSCC.

## Data Availability Statement

Publicly available datasets were analyzed in this study. This data can be found here: https://portal.gdc.cancer.gov/, https://www.ncbi.nlm.nih.gov/geo/, https://string-db.org/, http://www.mircode.org/, http://www.mirdb.org/, http://mirtarbase.mbc.nctu.edu.tw/, http://www.targetscan.org.

## Ethics Statement

The study protocol was approved by the Ethics Committees of Nanfang Hospital of Guangdong Province (NO: NFEC-2018-027).

## Author Contributions

GH: design and initiation of the study, quality control of data and algorithms, data analysis and interpretation, manuscript preparation and editing. QW: data acquisition. ZZ: statistical analysis. TS: patient recruitment and clinical support and oversight. X-ZL: study concept, design and initiation of the study. All authors are revision and approval the final version of the paper.

### Conflict of Interest

The authors declare that the research was conducted in the absence of any commercial or financial relationships that could be construed as a potential conflict of interest.

## Supplementary Material

Supplementary Figure 1 6 hub genes associated with overall survival. **(A)** GNA14, **(B)** GRM5, **(C)** (KRT3), **(D)** KRT26, **(E)** TRAC1, **(F)** HTR2C.

Supplementary Figure 2 Kaplan–Meier analysis along with logrank *p* was used to compare the survival of the low-risk group and high-risk group. **(A)** Survival analysis of training set. **(B)** Survival analysis of validation set. **(C)** OSCC patients with grade I and II. **(D)** OSCC patients with grade IV and III. **(E)** ROC based on risk score model.

Supplementary Figure 3 The mRNA expression level of DKK1 and HOXC6 in OSCC **(A)**. The protein level of CMA1 and GNA14 in OSCC cell lines and tissues **(B)**. Combination of two and more biomarkers predicted OSCC patients overall survival.

## Abbreviations

OSCC: oral squamous cell carcinoma
TCGA: The Cancer Genome Atlas
DERNAs: differentially expressed RNAs
DEmRNAs: differentially expressed mRNAs
DElncRNAs: differentially expressed lncRNAs
DEmiRNAs: differentially expressed miRNAs
GEO: Gene Expression Omnibus
ceRNA: competing endogenous RNA
PPI: protein-protein interaction
DMEM: Dulbecco's modified Eagle's medium
FBS: fetal bovine serum
MREs: miRNA response elements
ROC: receiver operating characteristic
GO: Gene ontology
KEGG: Kyoto Encyclopedia of Genes and Genomes
ncRNAs: non-cording RNAs.
3'-UTR: 3'-Untranslated region.
