# Supplementary figures and images for: Identification of Candidate Biomarkers and Analysis of Prognostic Values in Oral Squamous Cell Carcinoma
Source: Front Oncol. 2019 Oct 18;9:1054. doi: 10.3389/fonc.2019.01054 (PMC6813197; doi:10.3389/fonc.2019.01054)

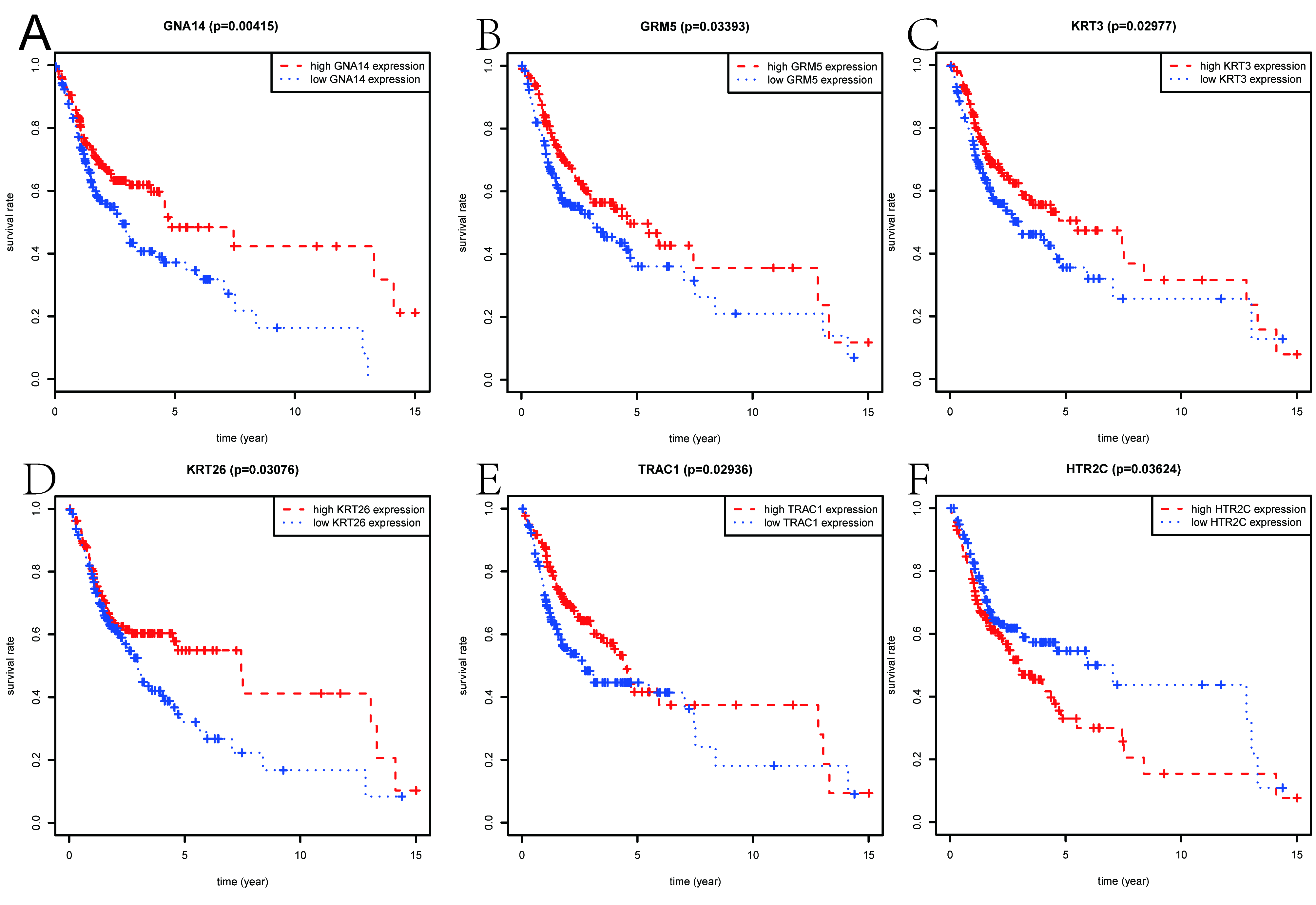

Supplement: Supplementary Figure 1 — 6 hub genes associated with overall survival. (A) GNA14, (B) GRM5, (C) (KRT3), (D) KRT26, (E) TRAC1, (F) HTR2C. [file Image_1.TIF]

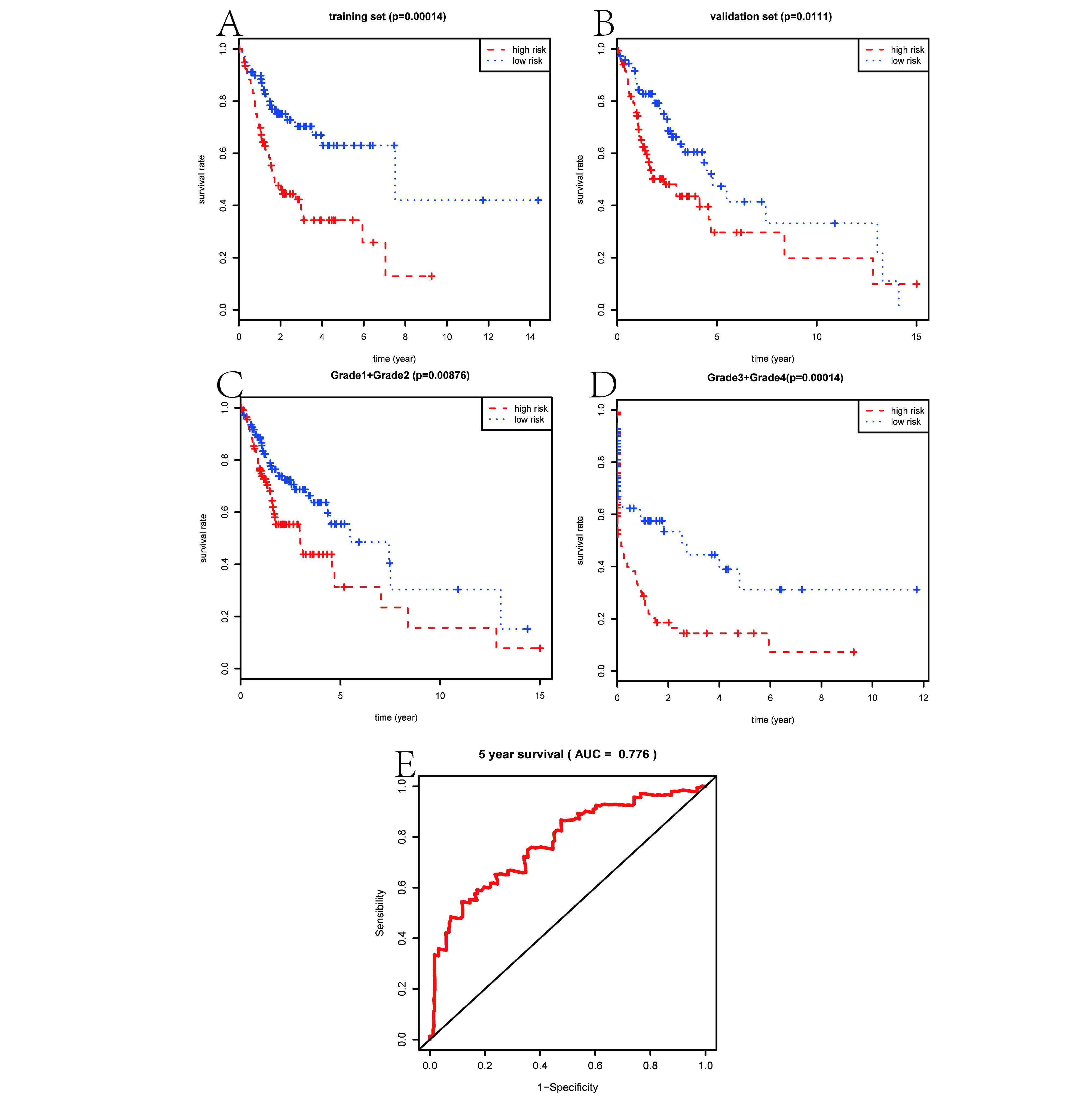

Supplement: Supplementary Figure 2 — Kaplan–Meier analysis along with logrank p was used to compare the survival of the low-risk group and high-risk group. (A) Survival analysis of training set. (B) Survival analysis of validation set. (C) OSCC patients with grade I and II. (D) OSCC patients with grade IV and III. (E) ROC based on risk score model. [file Image_2.TIF]

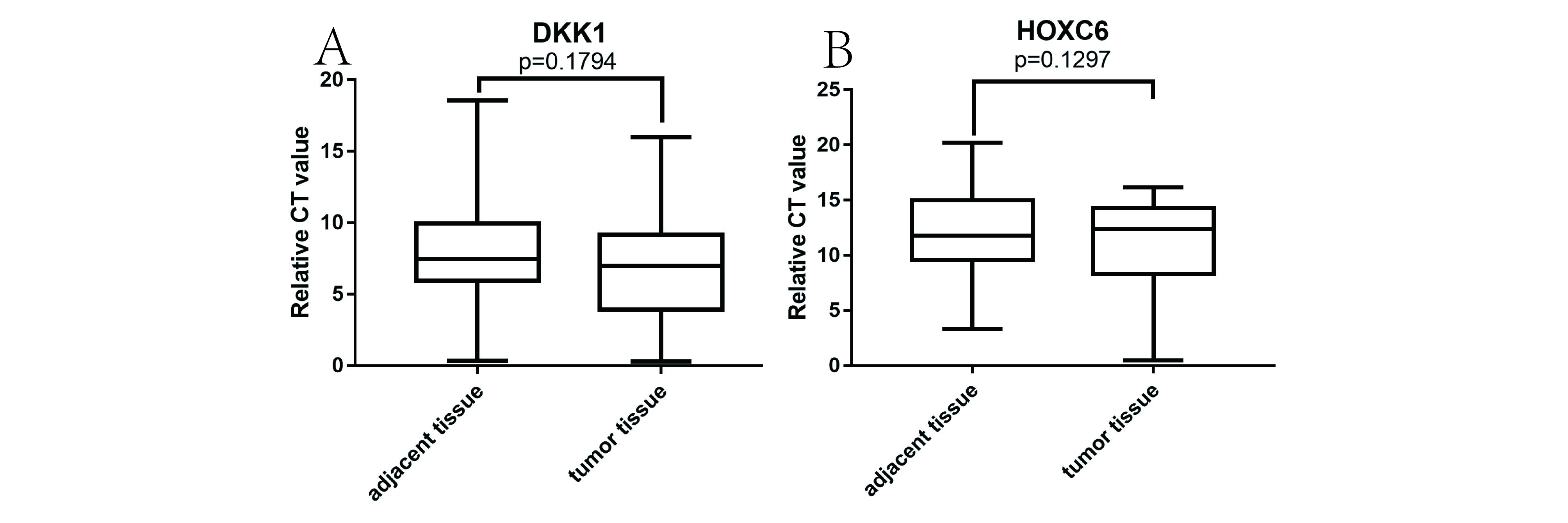

Supplement: Supplementary Figure 3 — The mRNA expression level of DKK1 and HOXC6 in OSCC (A). The protein level of CMA1 and GNA14 in OSCC cell lines and tissues (B). Combination of two and more biomarkers predicted OSCC patients overall survival. [file Image_3.TIF]
